# Professionalization trajectory of healthcare interpreter certification in the context of the language industry: a comparative study of industry-led and government-led models, with implications for China

**DOI:** 10.3389/fpubh.2025.1650602

**Published:** 2025-09-22

**Authors:** Wei Hu, Jiaming Jiang

**Affiliations:** ^1^School of International Business Communications, Dongbei University of Finance and Economics, Dalian, Liaoning, China; ^2^School of Information and Data Sciences, Nagasaki University, Nagasaki, Japan

**Keywords:** language industry, healthcare interpreting, certification system, professionalization trajectory, limited official language proficiency (LOP)

## Abstract

This study investigates the professionalization trajectory of healthcare interpreter certification under the emerging paradigm of the language industry and discusses the implications for China's nascent system. Against the backdrop of increasing linguistic diversity and growing demands for multilingual healthcare communication, two contrasting institutional models are compared: the industry-led model favored in the United States and the government-led model as developed in Japan. Drawing on professionalization theory and the political economy of language services, the paper explores how different actors—professional associations, state agencies, and language service providers—shape certification standards, ethical frameworks, and service integration mechanisms. The analysis highlights the differences between the two model types: while the U.S. model prioritizes decentralized standard-setting, market-driven legitimacy, and platform-based coordination, the Japanese model relies on centralized policy engineering, institutionalized ethics, and government-led service embedding. Building on this comparative framework, the paper proposes a dual-track approach to certification system development in China, combining top-down policy guidance with bottom-up industry collaboration. While this model offers a balanced pathway toward standardization and flexibility, it also entails potential risks such as institutional overlaps, uneven regional adoption, and challenges of long-term sustainability. It argues that healthcare interpreter certification should function not only as a regulatory mechanism but also as a strategic instrument for language governance, public health equity, and industry development. The study concludes by advocating for the integration of certification systems into China's national language strategy, emphasizing institutional sustainability, ethical accountability, and international competitiveness as key pillars of a future-oriented healthcare language service ecosystem.

## 1 Introduction

With the deepening of the digital economy and the globalization of the service sector, language services have extended well beyond traditional domains such as education and translation. They have gradually developed into a comprehensive language industry encompassing language intelligence, translation technologies, language education, and creative language applications. Globally, the language services industry has evolved into a multi-billion-dollar market, with healthcare, government, financial, and legal as some of the strongest growth verticals (1). Leading language service providers increasingly integrate digital platforms, AI-driven solutions, and multilingual communication services, while also facing persistent challenges of talent shortages, price pressures, and uneven adoption across regions. In China, this trend has been reinforced by the State Council's *Opinions on Comprehensively Strengthening Language and Script Work in the New Era* (2020), which explicitly calls for “strengthening planning and research on the language industry” and promoting a coordinated approach that combines “government guidance with market operation.” The emphasis was further underscored by Xi Jinping emphasizing the need to improve services and regulations that facilitate the daily life of foreigners living in China (2). This further signals China's strategic commitment to building an inclusive, service-oriented, and legally grounded environment for multilingual governance and international engagement.

Against this backdrop, healthcare interpreting has emerged as a particularly salient and complex subdomain within the language services industry, owing to its high degree of specialization, ethical sensitivity, and service-related risk. In clinical settings, healthcare interpreters function not only as linguistic mediators between patients and providers but also as critical agents influencing the accuracy of communication and the quality of clinical decision-making. Especially in countries experiencing growing immigrant populations and increasingly diverse patient language profiles, official language barriers in healthcare encounters have evolved from a mere technical inconvenience into a significant issue of public health and social equity. Yet industry data highlight a growing shortage of trained professionals, particularly in high-impact fields like healthcare (1). The institutionalization of healthcare language services thus represents a vital step toward improving healthcare delivery and safeguarding patient rights. In this context, the establishment of a healthcare interpreter certification system is essential not only for recognizing professional competencies but also for signaling the maturity of a nation's language service institutions and the degree of standardization within the industry.

In China, efforts to build healthcare language capacity are gaining momentum under national-level policy guidance. Pilot initiatives have been launched in Beijing, Shanghai, Sichuan, and Hainan to establish International Medical Centers. These programs aim to create multilingual service windows and foreign outpatient clinics within hospitals. For example, the Shanghai International Medical Center provides services in four languages—Chinese, English, Japanese, and Korean—while the Boao Lecheng International Medical Tourism Pilot Zone has developed an all-English medical environment with foreign physicians and bilingual nursing teams. At present, many top-tier hospitals offer English-language navigation, interpreter apps for diagnosis, and remote interpreting support. However, significant challenges remain in the areas of standardized training, unified certification mechanisms, and the uneven distribution of language services across cities and hospitals. As a result, the institutionalization, equity, and professionalization of China's medical language service system still require substantial improvement.

Despite the increasing relevance of healthcare interpreting, scholarly research has largely concentrated on operational practices (3, 4) and ethical role conceptualizations (5–7), with relatively little attention given to institutional development and industrial governance from a language policy perspective. This study shifts the analytical focus toward the language industry context. It asks: How do certification systems unfolds across different institutional models, and what mechanisms enable the construction of professional legitimacy in healthcare interpreting?

To answer these questions, the present study adopts Abbott's (8) theory of the “system of professions” and the notion of jurisdiction as its primary analytical framework. The framework is further informed by Evetts' (9) distinction between “professionality” and “professionalism,” and Noordegraaf's (10) concept of “hybrid professionalism.” Using these theoretical tools, the study conducts a comparative analysis of two contrasting national models: the industry-led model exemplified by the United States and the government-led model developed in Japan. For the purpose of this study, the term industry-led model refers to certification and standardization processes primarily shaped by industry associations, market demand, and professional bodies, whereas the government-led model refers to accreditation and institution-building primarily driven by national policy agendas and administrative leadership. It examines the institutional actors involved—professional associations, regulatory bodies, and language service providers (LSPs)—and analyzes their respective roles in shaping certification standards, ethical frameworks, and mechanisms for service integration.

The present study examines the professionalization trajectory of healthcare interpreter certification within the broader context of the language industry, thereby expanding the scope of inquiry beyond the micro-level concerns of interpreter roles and pedagogical training to include macro-level issues such as language policy, public governance, and language service infrastructure. It proposes the concept of a “dual-track certification model” as a theoretically grounded and policy-relevant recommendation tailored to the Chinese context. By bridging translation studies, public policy, and language economics, this study contributes a new perspective on how certification systems operate as instruments of professionalization, industry regulation, and public service delivery. The findings provide both a theoretical framework and actionable insights for policymakers, educators, and stakeholders in the language services industry.

## 2 Literature review

### 2.1 Theoretical framework of professionalization

The construction of occupational legitimacy and institutionalization has long been a central concern in the sociology of professions and institutional studies. The present study adopts Abbott's (8) theory of the “system of professions” as its analytical starting point. Abbott argues that professionalization is not the natural unfolding of intrinsic qualities, but rather a process of institutional construction in which professions assert jurisdiction over specific domains through claims to expert knowledge, control of work tasks, and state recognition. In the context of healthcare interpreting, the emergence of certification systems represents an effort to assert jurisdiction within the subfield of medical language services.

Building on this, Evetts (9) offered a helpful distinction between “professionality” and “professionalism.” The former refers to internally generated norms, ethics, and identity within professional communities, while the latter denotes external regulatory mechanisms imposed through organizations, markets, and state policies. This conceptual duality is particularly useful in analyzing how industry-led and government-led certification systems differ in their approaches to standard formation, ethical regulation, and institutional embedding.

Noordegraaf (10) added a further dimension by introducing the notion of “hybrid professionalism,” emphasizing the increasing interdependence of professional logic, organizational structures, and public accountability. In high-risk and policy-sensitive fields such as healthcare interpreting, professional identity is shaped not only by internal standards but also by institutional control and broader governance frameworks. Consequently, the professionalization process is often hybrid in nature—negotiating between autonomy and accountability, ethics and compliance, and market dynamics and public interest.

This presents constructs a comparative framework for analyzing healthcare interpreter certification by using Abbott's system of professions as the theoretical backbone, supported by Evetts' insights on dual regulatory logics and Noordegraaf's notion of hybrid professionalism. This layered framework enables a nuanced analysis of how professional legitimacy is negotiated across institutional contexts.

### 2.2 Research on healthcare interpreter certification systems

The professionalization of healthcare interpreting—marked by the increasing emphasis on expert knowledge, ethical conduct, and communicative competence—has attracted growing attention from both the academic and policy-making communities. This section surveys major strands of research related to certification development, role theory, and institutional implementation, setting the stage for a comparative institutional analysis.

Angelelli (5) introduced the concept of a “visibility continuum” to challenge the traditional view of interpreters as neutral conduits, instead emphasizing their active role in provider-patient interactions. Hsieh (6, 11) further developed the “coordinating agent” model, which highlights the interpreter's negotiation of overlapping roles and the conflicts arising from institutional expectations. These works have laid important foundations for understanding the interpreter's professional responsibilities and ethical positioning.

Research on certification systems has primarily focused on the development of standards, assessment mechanisms, and the struggle for professional legitimacy. Youdelman (12) conducted a comprehensive review of the U.S. system, identifying the establishment of the Certification Commission for Healthcare Interpreters (CCHI) and the National Board of Certification for Medical Interpreters (NBCMI) as pivotal milestones in the standardization of the field. She further examined how federal policies and civil rights legislation (e.g., Title VI) underpinned the emergence of professional standards.

In contrast, Japanese scholarship has emphasized institutional frameworks and policy pathways. Hu et al. (13) analyzed the guidelines of the Ministry of Health, Labour and Welfare (MHLW) and the development of the Japan Medical Service Accreditation for International Patients (JMIP), highlighting the central role of government-led initiatives in shaping certification standards and service delivery models.

Recent studies have also explored training programs and quasi-certification systems (i.e., structured yet non-legally mandated models), including those developed in Turkey (14) and South Korea (15). However, comparative analyses of how institutional models shape certification systems remain relatively underexplored. In particular, few studies have addressed how professional legitimacy, ethical frameworks, and service delivery mechanisms are co-constructed across different governance regimes. The present study seeks to fill that analytical void by introducing the concept of a “professionalization trajectory” as a basis for comparing the U.S. and Japanese models, with implications for emerging systems such as China's.

### 2.3 Interpreting as a profession through the lens of the language industry

The concept of the “language industry” was first introduced by policy institutions such as the European Language Industry Association to underscore the economic and strategic value of language-related services. In recent years, this concept has gained traction in China, becoming part of the national language strategy (16). The Ministry of Education of the People's Republic of China (17) has stressed the importance of building the language service industry into a key pillar of the national digital economy, with particular emphasis on integration with healthcare, education, and AI-driven innovation. This vision further supports the institutional embedding of interpreting professions within broader industrial development agendas.

From the perspective of language economics, interpreting constitutes a knowledge-intensive service sector within the value chain of the language industry (18). Scholars such as Pym et al. (19) have emphasized the role of market structures and digital platforms in reshaping professional boundaries. Although these studies highlight the institutional embeddedness of interpreting in broader economic systems, relatively few have addressed the role of certification as an instrument of industrial governance. This is particularly important in the case of healthcare interpreting, which requires high ethical standards, technical accuracy, and organizational coordination. Recent industry data indicate that interpreting is increasingly incorporated into national infrastructure strategies, particularly in public health and emergency services (20).

By situating healthcare interpreter certification within the evolving architecture of the language industry, this study moves beyond traditional frameworks of “professional ethics and training” toward a more comprehensive perspective of “policy alignment, industrial integration, and systemic innovation.” This shift in perspective positions certification not merely as a professional credential but as an instrument of industrial governance and public service modernization.

## 3 Methodology

### 3.1 Data sources and selection criteria

This study draws on a combination of academic literature, government policy documents, industry reports, and statistical yearbooks. Databases consulted include CNKI, Web of Science, CiNii, and official portals of the Ministry of Health, Labour and Welfare (Japan) and the U.S. Department of Health and Human Services. The literature search covered the period from 2000 to 2025, using keywords such as “healthcare interpreting,” “medical interpreting,” “certification system,” “professionalization,” and “language industry.” Inclusion criteria comprised peer-reviewed publications, publicly available policy documents, and official statistical data. Exclusion criteria involved unpublished drafts, opinion pieces lacking empirical support, and data without clear methodological disclosure. Secondary data were thematically coded in three stages:

1. Open coding: Initial reading of all collected materials to identify recurring concepts and issues related to healthcare interpreter certification, such as governance structure, ethical frameworks, and service integration mechanisms. Codes were assigned directly to text segments without predefined categories.
2. Axial coding: Grouping of open codes into broader thematic categories, guided by Abbott's theory of professions, Evetts' professionality/professionalism distinction, and Noordegraaf's hybrid professionalism concept. For example, codes on “market validation” and “professional association standards” were merged into the theme “market-driven legitimacy formation.”
3. Cross-case synthesis: Comparative examination of U.S. and Japanese themes to identify convergences and divergences. For instance, the “centralized” policy alignment theme in Japan was contrasted with the “decentralized” market coordination theme in the U.S., with illustrative examples from policy documents and adoption statistics.

### 3.2 Limitations of secondary data source

The study relies primarily on secondary data, whose accuracy and representativeness are contingent on the methodologies of the original sources. Official statistics, such as JMIP awareness rates among medical institutions reported in national surveys, may under- or overestimate actual implementation due to limited sample coverage, self-reported responses, and the possibility that smaller institutions are underrepresented or misclassify their level of compliance. To mitigate potential biases, key statistics were cross-checked with alternative sources where feasible. However, for certain large-scale surveys of medical institutions—such as those assessing JMIP awareness—no third-party datasets were identified, likely due to ethical constraints and the difficulty of accessing institutional-level information.

## 4 Industry-led pathways: market-driven professionalization in the U.S.

### 4.1 Emergence of professional roles under market mechanisms

This section traces the initial emergence of healthcare interpreting as a professional role in the United States, highlighting how market demand catalyzed community-based responses and gradually led to professional role formation. From the 1980s to the early 1990s, the U.S. healthcare system lacked systematic language support services for patients with limited official language proficiency (LOP). It was common for hospitals to depend on untrained individuals such as family members, administrative staff, or medical students to provide *ad hoc* interpreting. These informal practices resulted in inconsistent communication quality, frequent ethical breaches, and serious clinical risks.

Several high-profile incidents revealed the dangers of non-professional interpreting. For example, due to the mistranslation of the Spanish word *intoxicado*, a patient was misdiagnosed with alcohol intoxication, when in fact the patient had suffered a brain hemorrhage (21). Other cases have involved medical personnel or custodial staff interpreting without training, leading to miscommunication and compromised patient safety (12).

In response, hospitals, non-profit organizations, and community clinics began appointing language service coordinators and offering basic training for bilingual staff. Volunteer interpreter programs and multilingual hotlines emerged in urban areas, and commercial LSPs (e.g., Language Line Services) expanded into healthcare sectors. While these early efforts varied in quality and structure, they reflected growing market recognition of interpreting as a distinct and necessary professional role.

This initial phase marked a transition from fragmented, informal interpreting to the gradual development of role clarity, ethical expectations, and institutional visibility. As legal and practical pressures intensified, healthcare interpreting evolved from an invisible support function into an emergent profession shaped by service needs and market incentives.

### 4.2 Industry-led standard development

The early 2000s saw the formalization of healthcare interpreting in the U.S. through standard-setting initiatives led by professional associations and non-governmental actors. Although federal policies such as Title VI of the Civil Rights Act and the CLAS (Culturally and Linguistically Appropriate Services) Standards provided a regulatory foundation, the government did not establish a centralized certification authority. Instead, civil society and industry actors assumed the primary role in developing assessment standards and establishing institutional legitimacy.

Two major organizations spearheaded this effort. The Certification Commission for Healthcare Interpreters (CCHI), launched in 2009, introduced the CoreCHI™ and CHI™ credentials, with broad language coverage and a focus on general competencies. Simultaneously, the National Board of Certification for Medical Interpreters (NBCMI), supported by the International Medical Interpreters Association (IMIA), offered CMI™ certification targeting high-demand languages and emphasizing medical specialization.

These certifications were a response to institutional and patient demands for qualified language support and were widely adopted by LSPs. Major companies such as Language Line Solutions and CyraCom International list certification as a preferred or required credential, integrating it into recruitment, training, and service evaluation processes. Certain LSPs also participate directly in certification implementation, serving as intermediaries between assessment systems and employment markets.

From the perspective of professionalization theory, this model illustrates Evetts' (9) concept of coordinated interaction between internally defined professional norms (“professionality”) and externally imposed regulatory structures (“professionalism”). Despite lacking formal state endorsement, the U.S. system has achieved a high level of de facto professional legitimacy through bottom-up institutionalization and industry validation.

### 4.3 Platform-based integration

As healthcare interpreter certification matured, digital platforms emerged as key enablers of system expansion, accessibility, and quality assurance. This section examines how technology has facilitated scalable certification practices and supported the development of a cohesive professional ecosystem.

Testing service providers such as Pearson VUE and ProctorU became essential partners of CCHI and NBCMI, administering exams, managing test content, and ensuring data integrity and security. CCHI offers its exams through Pearson VUE's computer-based testing network, while NBCMI uses ProctorU's remote proctoring platform to deliver home-based assessments. These systems incorporate biometric identity verification and anti-cheating protocols, enhancing the credibility, and transparency of the certification process.

Continuing education units (CEUs) are also managed via online platforms, allowing certified interpreters to track renewal requirements and engage in ongoing professional development. Since 2023, CCHI has piloted an AI-assisted scoring system (e.g., speech analysis, semantic accuracy detection) to assess interpreting performance, particularly in oral scenarios. This innovation addresses evaluation bottlenecks and demonstrates how language technologies increasingly influence assessment design.

Feedback from LSPs has shaped certification content, especially in areas such as telehealth, emergency services, and cultural mediation. Certification frameworks have evolved in response to changing service contexts, showcasing a dynamic interaction between standard-setting bodies, industry needs, and technological tools. This ongoing integration positions digital platforms not just as delivery mechanisms but as co-architects of the professional infrastructure.

### 4.4 Summary: professionalization through decentralization and industrial integration

The U.S. pathway to healthcare interpreter professionalization is characterized by decentralized governance, market-driven legitimacy formation, and digital ecosystem integration. Certification standards are set by professional associations, operationalized by testing platforms, and validated by language service providers. This model enables flexible coordination across sectors while embedding ethics, competencies, and service alignment into a stable institutional framework.

This approach exemplifies Abbott's (8) theory of jurisdiction, wherein professional groups establish authority over specific domains by asserting expert knowledge and integrating service functions. Although the U.S. model lacks formal legal mandates, it has achieved wide recognition and sustainable operation through voluntary participation and market-based coordination.

The U.S. system represents an institutional trajectory—one grounded in civil society initiative, private sector engagement, and platform-based professionalization. This offers a valuable reference point for countries seeking to develop healthcare language services through adaptive, industry-led mechanisms.

## 5 Government-led pathways: policy-driven professionalization in Japan

### 5.1 Role formation under policy supervision

Unlike the industry-led emergence observed in the U.S., the professionalization of healthcare interpreting in Japan was not initiated by market demand or civil society organizations. Instead, the early development was shaped by top-down policy attention and government-supervised experimentation. Prior to the mid-2000s, Japan lacked a defined institutional framework for the accommodation of foreign patients in healthcare settings. Medical facilities often relied on *ad hoc* solutions, such as bilingual administrative staff or volunteer interpreters, and language barriers were treated as logistical rather than professional issues.

The turning point came with the Japanese government's strategic recognition of medical services for foreign nationals as part of national policy. This awareness was linked not only to the aging population and labor market shifts but also to tourism promotion and soft-power diplomacy. In this context, the Ministry of Health, Labour and Welfare (MHLW) initiated pilot initiatives funded through public subsidies to support multilingual service readiness in selected hospitals. These programs encouraged hospitals to experiment with signage translation, appointment interpretation, and the hiring of multilingual staff.

Despite the introduction of these initiatives, interpreter training, and credentialing remained informal. The government primarily played a supervisory role, allowing hospitals to experiment within loose frameworks. Nevertheless, these efforts demonstrated an early recognition of medical interpreting as a necessary function of healthcare access, and framed language support as a measurable element of public service performance.

This phase laid the policy foundation for institutionalizing healthcare interpreting in Japan. While role formation was slow and fragmented, it was guided by governmental priorities rather than bottom-up professional initiative, which shaped the direction of subsequent standardization and integration efforts.

### 5.2 Government-orchestrated standardization

The second stage of professionalization was defined by the state's efforts to disseminate service standards and promote institutional accountability. Around 2010, Japan's medical language services underwent a pivotal transition from locally driven practices to centrally coordinated institutional engineering. This policy shift can be traced back to the “New Growth Strategy” adopted by the Democratic Party administration (under Prime Minister Naoto Kan) in 2010, which identified “tourism-oriented nationhood,” “international medical exchange,” and “job creation” as key national development priorities. The strategy explicitly called for the establishment of an “environment for accepting international patients” and incorporated language services as a core component in enhancing Japan's capacity for international medical services.

To implement this strategy, the MHLW launched the Support Project for the Development of the JMIP in 2011 (39). The project aimed to promote the systematic development of multilingual language service capabilities in healthcare institutions through centralized planning. It explicitly set “improving the quality of medical services” as its overarching goal and proposed the establishment of a nationally unified hospital accreditation framework, with “interpreter support systems” designated as one of the core evaluation criteria. This policy marked the first time that medical language services were incorporated into a national institutional governance framework in Japan, reflecting a high degree of policy integration and systemic design.

In April 2011, following a public bidding process, the MHLW selected Nichii Gakkan Co., Ltd. as the implementing agency responsible for developing the overall structure, evaluation mechanisms, and operational procedures of the certification system. Nichii was also tasked with drafting standards for interpreter deployment and service quality. This institutional framework later became the foundation for the JMIP system, and its design logic embodied a quasi-market model in which state-defined standards were implemented by third-party organizations.

Meanwhile, local governments, under central guidance, began launching interpreter training programs tailored to regional needs. These programs included curriculum development, assessment design, and exploratory linkages with local employment initiatives. Medical interpreting gradually evolved from a form of voluntary language support into a semi-professionalized service with preliminary certification mechanisms. Its legitimacy as a profession was no longer derived solely from social recognition or institutional demand but was increasingly secured through centralized policy support and institutionalized professionalization pathways.

### 5.3 Standard diffusion and the formation of a quasi-certification framework

As the JMIP framework expanded nationwide and local-level training programs advanced, Japan's medical language service system entered a new stage marked by the integration of standards and the refinement of quasi-certification mechanisms. This phase was characterized by the gradual extension of centrally defined institutional frameworks to regional levels, and the transformation of implementation practices from a training-oriented approach to a competency assessment–based service access model. The professionalization of medical interpreting began to take on a quasi-certification structure.

At the level of training systems, although the Ministry of Health, Labour and Welfare did not issue legally binding national curriculum mandates, a de facto “national training benchmark” emerged through the development of instructional materials and model programs by the National Center for Global Health and Medicine (NCGM). These central-level guidelines were widely adopted by local governments, international exchange associations, medical societies, and non-profit organizations to carry out standardized training programs supported by public funding. Most training courses comprised 80 to 120 h of modules covering such topics as medical terminology, ethical norms, situational communication, and field-based practicums. Completion of the training was typically followed by competency testing. The institutionalization of such training not only enhanced the professionalism of language services but also introduced a quasi-threshold effect at the point of entry into the profession.

On the certification side, the Japan Medical Education Foundation (JME)—the operating body of the JMIP system—gradually expanded its role starting in 2015. In addition to overseeing institutional accreditation, it began promoting individual interpreter certification through the implementation of professional qualification exams. Its “Medical Interpreter Skills Certification Exam” (Iryo Tsuyaku Senmon Gino Nintei Shiken) has steadily gained credibility nationwide and is now widely referenced by healthcare institutions when hiring interpreters. The exam evaluates a broad range of competencies, including ethical reasoning, foundational medical knowledge, terminology, interpreting techniques, and situational expression. Performance on the exam directly influences whether interpreters can be officially employed by hospitals, government departments, or service platforms.

According to *Results of the Survey on the Acceptance of Foreign Patients in Medical Institutions* (FY2018–2023) (22–27), which constitute all available data since the survey's launch in 2018 (first published in 2019), the number of JMIP-certified institutions remained very limited throughout the 6-year period. The peak was reported in FY2019, with 63 institutions, but subsequent years did not show consistent growth. In some years, the figure even declined compared with the previous year, which may be partly attributed to fluctuations in the recovery rate of Questionnaire A, the primary survey instrument. Overall, despite annual surveys covering over 5,000 institutions, the adoption rate has stagnated at around 1%, indicating that JMIP dissemination has failed to expand meaningfully.

In contrast, demand-side indicators demonstrate steady or even accelerated growth. The number of foreign residents increased from 2.73 million in 2018 (2.16% of the total population) to 3.41 million in 2023 (2.74%), representing a 25% net increase despite temporary declines in 2020–2021 during the COVID-19 pandemic (28). Meanwhile, inbound tourist arrivals reached 31.9 million in 2019 before collapsing to 0.25 million in 2021, then rebounded sharply to 25.1 million by 2023 (29).

This mismatch reveals a structural lag: while both long-term and short-term foreign patient populations have expanded significantly over the last decade, the institutional uptake of JMIP has plateaued, failing to respond adequately to the heightened and diversified demand for multilingual healthcare access.

From the perspective of language industry development, this stage represents a critical leap from “standardized training” to a “certification-based professional system” for medical language services in Japan. Certification no longer functions merely as a symbolic marker of quality but rather serves as a central coordination mechanism connecting language service supply with the institutional demands of the healthcare system. During this period, the medical interpreting profession gained increasing institutional embeddedness—not only shaping the professional development of individuals but also influencing organizational operations and quality assurance mechanisms within service systems.

### 5.4 Summary: professionalization through centralized governance and policy alignment

Japan's pathway to healthcare interpreter professionalization can be summarized as a policy-driven and institutionally orchestrated model. Role formation was initiated under government supervision, professional standards were the product of accreditation-based incentives, and service integration was achieved through cross-sectoral coordination and digital infrastructure. This trajectory contrasts sharply with the U.S. model, which relied on decentralized certification and industry-led standard development.

Rather than formalizing interpreting as an autonomous profession, the Japanese approach embedded interpreting into public service delivery systems and administrative accountability regimes. The logic of legitimacy was institutional rather than occupational, emphasizing systemic coordination over professional independence.

This model exhibits clear strengths: it ensures alignment with national policy priorities, facilitates large-scale implementation through public funding, and enables cross-sectoral synergy. However, it may also limit the emergence of interpreter-led professional communities and constrain innovation due to its top-down orientation.

The Japanese case thus highlights an alternative route to professionalization—one that substitutes regulatory enforcement with performance governance, and market competition with policy coordination. This perspective informs the comparative discussion that follows, offering insights into how different governance logics shape the institutional trajectories of healthcare interpreter certification systems.

## 6 Institutional comparison and developmental implications for China

### 6.1 Comparative analysis of professionalization paths

Building on the analytical framework established in Sections 4 and 5, this section systematically compares the two professionalization trajectories of healthcare interpreter certification in the United States and Japan. These models exhibit systematic differences in institutional leadership, legitimacy formation, ethical regulation, service integration, and theoretical foundations, each demonstrating distinct strengths, and limitations. To present these contrasts more clearly, Table 1 summarizes the key features of the two models across five analytical dimensions, along with their respective strengths and limitations.

**Table 1:** Comparative features of U.S. and Japan models.

**Dimension**	**U.S. (Industry-led)**	**Japan (Government-led)**
Leadership	Driven by professional associations (CCHI, NBCMI); bottom-up.	Coordinated by Ministry of Health, Labour and Welfare; top-down.
Legitimacy	Market validation through employer recognition and client trust; credentials preferred but not mandatory.	Policy mandates and hospital accreditation confer legitimacy; interpreters subsumed under institutional compliance.
Ethics	Scenario-based training, competence-focused, assessed in certification exams.	Standardized ethical modules designed by central agencies; emphasis on normative compliance.
Service integration	Directly tied to employment, insurance reimbursement, and remote interpreting platforms.	Embedded in accreditation, emergency preparedness, and tourism-related packages.
Theoretical framing	Reflects Abbott's (8) “jurisdiction” model: autonomy, competition, peer-based claims.	Resonates with Noordegraaf's (10) “institutionalized professionalism”: policy alignment, bureaucratic accountability.
Strengths	Flexible, responsive to market demand, fosters innovation, and professional autonomy.	Ensures consistency, systemic coherence, institutional legitimacy, and policy integration.
Limitations	Fragmentation, uneven adoption, lack of unified authority.	Rigidity, limited professional autonomy, weaker market responsiveness.

The industry-led model exemplified by the U.S. demonstrates notable advantages, particularly its flexibility and market responsiveness. Certification processes initiated by industry bodies such as CCHI and NBCMI closely align credentialing standards, assessments, and services with market demands. This model allows healthcare interpreting services to rapidly adjust to multilingual and varied healthcare contexts. Integration with employment criteria in hospitals, insurance reimbursement schemes, and remote interpreting platforms creates a comprehensive “assessment–employment–payment” loop, enhancing the practical value and employability of certified interpreters. Moreover, ethical competence training, delivered through scenario-based simulations embedded within certification curricula, effectively prepares interpreters for complex real-world situations. The legitimacy derived from market acceptance and institutional adoption further underscores its decentralized, competence-based approach.

Despite these advantages, the industry-led approach faces significant challenges. Without mandatory national standards or governmental enforcement, there is considerable variability in how certification standards are adopted regionally and institutionally, leading to potential fragmentation. The coexistence of multiple certification entities, while encouraging competition and innovation, may also produce confusion over standards and dilute the profession's clarity and cohesion.

In contrast, the government-led model seen in Japan offers substantial strengths related to policy mobilization and standard integration. Supervised directly by the Ministry of Health, Labour and Welfare or administered through designated third-party organizations, certification frameworks such as the JMIP and interpreter qualification examinations ensure uniformity and consistency across training, assessment, and service provision. Ethical training, disseminated through centrally developed modules, guarantees standardized understanding and implementation of professional guidelines. Additionally, embedding interpreter certification into hospital accreditation, disaster response frameworks, and international patient services firmly situates interpreters within public service and governance structures, solidifying their institutional legitimacy and role clarity.

Nevertheless, the Japanese model also reveals limitations, particularly concerning institutional rigidity and delayed market responsiveness. While standards are consistent and authoritative, they are not universally mandated as criteria for employment, leading to unclear career trajectories and weaker market integration. Moreover, the absence of professional autonomy and industry participation diminishes interpreters' voice in shaping standards, reducing the system's adaptability and responsiveness to rapidly changing service needs. Although standardized ethics training ensures uniform professional norms, it often neglects nuanced practitioner decision-making and on-site role conflicts, limiting practical effectiveness.

In sum, while the industry-led U.S. model excels in adaptability, innovation, and professional autonomy, it struggles with authoritative enforcement and standard uniformity. Conversely, Japan's government-led model effectively ensures consistency, systemic coherence, and public accountability, yet falls short in terms of flexibility, professional autonomy, and market-driven responsiveness. This complementarity provides theoretical insights valuable for exploring a hybrid “market-governance” pathway suited to countries like China, which seek a balanced approach in professionalizing healthcare language services.

### 6.2 Foundational conditions, feasibility, and risks of a dual-track model in China

#### 6.2.1 Foundational conditions

Regarding the resident foreign population, the *Seventh National Population Census Bulletin* issued by the National Bureau of Statistics indicates that there were approximately 845,000 foreign nationals living in China, primarily concentrated in major cities such as Beijing, Shanghai, Guangzhou, Shenzhen, and Hangzhou (38). The top source countries include South Korea, the United States, Japan, Myanmar, and Vietnam. Additionally, according to *Statistics on International Students in China in 2018* issued by the Ministry of Education (30), the number of international students in China reached 492,185 in 2018. The top 10 source countries for international students were South Korea, Thailand, Pakistan, India, the United States, Russia, Indonesia, Laos, Japan, Kazakhstan, and Vietnam. With the easing of post-pandemic entry restrictions, the frequency of foreign national activities in China has gradually recovered, leading to a rapid increase in demand for services, including a wide range of languages on medical services. As these services become increasingly integrated into the delivery of culturally competent and patient-centered healthcare, there is a growing need to establish formal systems for assessing interpreter competence. This is essential to prevent medical harm, ensure effective communication (12), and provide truly patient-centered care.

Unlike countries with a long history of immigration, China is facing the challenge of having a substantial number of foreign residents for the first time in recent years and needs to break from its previous monolingual (Mandarin) tradition (31). As the world's most populous developing country, China is undergoing a critical phase of transformation in public health internationalization, growing foreign resident populations, and the gradual emergence of a multilingual society. The national policy landscape increasingly prioritizes language services.

Although a national certification system for healthcare interpreting is not yet in place, a number of foundational elements are already visible across China. These include localized pilot programs, grassroots training initiatives, academic research in medical interpreting, and international healthcare platform development. However, it is important to note that, as of now, no comprehensive national statistics on healthcare interpreting or medical translation services have been publicly released through official channels such as government websites or major state-run media (e.g., *People's Daily, Xinhua News Agency*, or *China Daily*). Nevertheless, specific institutional cases highlight the scale and multilingual demand of such services. For example, United Family Healthcare (UFH), one of the country's largest private healthcare networks, operates 11 hospitals and 24 clinics nationwide. It serves more than one million patients annually, including nearly 100,000 foreign nationals. English accounts for over 50% of its internal clinical documentation, with additional support in languages such as Spanish and French (32). These examples indicate a growing need for professional healthcare interpreters and underscore the potential for building a more systematic certification ecosystem.

#### 6.2.2 Feasibility assessment

Drawing from the preceding comparative analysis, this section argues that China's future certification system should be guided by a dual-track model grounded in both language industry development and institutional capacity building. This model should feature strong government coordination while encouraging professional association and market participation, thereby enabling both standardization and adaptability. Accordingly, the following five key policy recommendations are offered:

1. Establish a dual-track system combining government leadership and industry collaboration. Inspired by Japan's policy mobilization logic, China could integrate medical language services into the national language strategy. At the same time, it should actively engage language service enterprises, academic institutions, industry associations, and healthcare organizations to foster a hybrid governance structure.
2. Phase in the certification system by leveraging regional pilot projects. Drawing on U.S. experience, China could initially support localized and voluntary certification initiatives led by professional communities, gradually building legitimacy and public awareness before integrating these systems into a broader national framework.
3. Center ethics and risk awareness in training and assessment. Building on the JMIP models, China should place ethics and patient safety at the core of its training modules and certification assessments. A context-sensitive ethical framework should be developed, aligned with China's legal and medical environments.
4. Ensure multilingual and differentiated service capability. Given China's unique linguistic landscape—spanning the language demands of the Belt and Road Initiative, cross-border communication, and minority languages—modular training and assessment frameworks, remote certification tools, and tiered language qualification standards should be adopted to improve service inclusivity and coverage. It is essential to develop differentiated training pathways based on varying levels of language demand. For example, tailored curricula should be designed for high-demand languages such as English, Russian, and Arabic, as well as for medium- and low-demand languages such as Uyghur.
5. Authorize third-party institutions to implement certification and training. To enhance credibility and capacity, qualified universities, language service companies, and research centers should be empowered to serve as certification and training providers, enabling a virtuous interaction between state oversight and professional expertise.

This dual-track approach envisions a certification system that is not merely an occupational qualification process but is also a policy instrument linking education, standards, service delivery, and industrial development. Ultimately, it can transition China's medical language services from *ad hoc* volunteerism to a sustainable, institutionalized profession embedded in national strategy.

#### 6.2.3 Potential risks and challenges

While the dual-track approach offers a balance between standardization and flexibility, potential risks should be acknowledged. These include:

1. Institutional risks: Possible conflicts between state regulatory frameworks and market responsiveness, unclear delineation of authority, and overlapping responsibilities.
2. Operational risks: Administrative burdens associated with dual governance, uneven adoption across regions, and disparities in resource allocation.
3. Socio-political risks: Resistance from professional communities, varying levels of stakeholder engagement, and challenges in aligning public expectations.

The Canadian case exemplifies these risks. Although Arya et al. (33) do not explicitly frame their analysis in terms of professionalization pathways, they reveal a hybrid pattern: some provinces fund telephone interpreting or maintain provincial interpreter banks, yet these efforts lack stable standards and long-term funding. At the same time, hospitals, community health centers, NGOs, and private actors attempt to fill gaps through *ad hoc* initiatives. This dual-track arrangement, while offering flexibility, leads to fragmentation, uneven regional access, and precarious resource bases that ultimately hinder the sustainable professionalization of medical interpreting services.

In Ontario, the absence of a unified provincial framework—unlike in British Columbia or Alberta where centralized systems are coordinated—has produced uneven standards and accountability gaps across institutions. Although the Healthcare Interpretation Network issued the National Standard Guide for Community Interpreting Services in 2007, the lack of a national certifying body means hospitals often fall back on divergent internal criteria, generating inconsistencies in interpreter qualifications and practice (34, 35). Moreover, the absence of standardized data collection across Local Health Integration Networks (LHINs) hampers systematic monitoring of language needs and limits evidence-based planning for service provision (36). Finally, disparities persist across departments and institutions in the actual uptake of interpreting services, reflecting uneven levels of awareness and training among clinical staff (37).

In sum, the Canadian experience demonstrates that while dual-track arrangements can offer short-term adaptability, the lack of coherent governance, unified standards, and sustainable funding mechanisms generates systemic vulnerabilities. Without addressing these risks, professionalization remains fragile, and the integration of medical interpreting into broader health policy frameworks is constrained. This underscores the need for more robust institutional design and stakeholder coordination.

## 7 Conclusion

In an era of growing global mobility and increasing linguistic diversity, language services have become an indispensable component of national governance, particularly in the healthcare sector. In this sector, language is not simply a communicative tool, it is vital to ensuring patient safety, health equity, and human dignity. Healthcare interpreting, as a highly specialized and ethically sensitive profession, stands at the intersection of public health, human rights, and language policy.

The present study compared the professionalization trajectory of healthcare interpreter certification systems. Through analysis of the industry-led model in the United States and the government-led model in Japan, it explored how different institutional logics—market-driven vs. policy-driven—shape certification practices, ethical frameworks, and service delivery mechanisms. These models demonstrate the diverse pathways through which professional legitimacy can be established, either through decentralized peer coordination or through centralized policy engineering.

By introducing a comparative framework grounded in the sociology of professions, the present study moves beyond descriptive accounts of interpreter roles and training programs to systematically analyze how certification systems operate as institutional mechanisms of professional control, standardization, and legitimacy formation. This approach offers a novel lens for examining how healthcare interpreting can be integrated into national service infrastructures under the broader umbrella of the language industry.

Against this backdrop, China is currently entering a crucial phase in the development of its language service infrastructure. The concept of the “language industry” has expanded beyond traditional education and cultural domains to encompass translation technology, AI-enabled services, and public service delivery. Within this evolving context, establishing a robust healthcare interpreter certification system is not merely a matter of standardization, but a strategic move toward building national language capacity and modernizing service systems.

This paper makes three key contributions. First, it anchors healthcare interpreter certification within the macro policy frame of the language industry, thereby bridging translation studies and language policy. Second, it shows the construction of a dual-pathway analytical model to compare industry-led and government-led certification systems, offering a structured reference for future institutional design. Third, it proposes the concept of a dual-track certification mechanism that accommodates both state regulation and industry participation—a policy-relevant contribution tailored to China's current reform priorities. These contributions extend the field of interpreter studies toward interdisciplinary integration with governance studies, labor regulation, and public service innovation.

As argued in this paper, the certification of healthcare interpreters should serve as both a professional regulation mechanism and a policy lever embedded in broader language governance. It can foster alignment between educational systems, industry standards, and public service needs. More importantly, it holds the potential to enhance China's international engagement, contribute to its Healthy China strategy, and support the construction of a high-quality, professionalized, and ethically grounded healthcare language service system.

Looking forward, China's pathway will need to balance state leadership with industry participation, ensuring that certification mechanisms are not only rigorous and inclusive, but also responsive to the diverse linguistic realities on the ground. The insights drawn from international models offer useful reference points, but the path ahead must ultimately reflect China's own developmental conditions and policy priorities.

The study relies primarily on secondary data. Still, limitations remain, and future research should incorporate primary data collection, such as interviews with stakeholders and field observations, to enhance empirical robustness.

## Data Availability

The original contributions presented in the study are included in the article/supplementary material, further inquiries can be directed to the corresponding author.
